# Gaps in hypertension and diabetes treatment among people living with and without HIV: Findings from a prospective cohort study in Kenya, Nigeria, Tanzania, and Uganda, 2013–2023

**DOI:** 10.1371/journal.pgph.0004464

**Published:** 2025-04-29

**Authors:** Matthew L. Romo, Nicole Dear, Trevor A. Crowell, Seth Frndak, Ajay P. Parikh, Hannah Kibuuka, John Owuoth, Valentine Sing’oei, Jonah Maswai, Emmanuel Bahemana, Victor Anyebe, Zahra Parker, Julie A. Ake, J. Sean Cavanaugh, Neha Shah, on behalf of the African Cohort Study (AFRICOS) Group

**Affiliations:** 1 U.S. Military HIV Research Program, CIDR, Walter Reed Army Institute of Research, Silver Spring, Maryland, United States of America; 2 Henry M. Jackson Foundation for the Advancement of Military Medicine, Inc., Bethesda, Maryland, United States of America; 3 Makerere University Walter Reed Project, Kampala, Uganda; 4 U.S. Military HIV Research Program, Walter Reed Army Institute of Research Africa, Kisumu, Kenya; 5 HJF Medical Research International, Kisumu, Kenya; 6 U.S. Military HIV Research Program, Walter Reed Army Institute of Research Africa, Kericho, Kenya; 7 HJF Medical Research International, Kericho, Kenya; 8 U.S. Military HIV Research Program, Walter Reed Army Institute of Research Africa, Mbeya, Tanzania; 9 HJF Medical Research International, Mbeya, Tanzania; 10 U.S. Military HIV Research Program, Walter Reed Army Institute of Research Africa, Abuja, Nigeria; 11 HJF Medical Research International, Abuja, Nigeria; 12 U.S. Military HIV Research Program, Walter Reed Army Institute of Research Africa, Lagos, Nigeria; University of California Irvine, UNITED STATES OF AMERICA

## Abstract

Hypertension and diabetes are increasingly important contributors to morbidity and mortality in African countries, including among people living with HIV (PLWH) who are on lifelong antiretroviral therapy. However, local HIV care programs have historically not included management of noncommunicable diseases. The African Cohort Study enrolls PLWH and people living without HIV (PLWoH) aged 15 years and older who are receiving clinical care at 12 PEPFAR-supported health facilities in Kenya, Nigeria, Tanzania, and Uganda. Participants undergo clinical assessments and medical record review every six months. We defined hypertension as a persistently elevated systolic and/or diastolic blood pressure ≥140/90 mmHg at two consecutive 6-monthly visits or receipt of hypertension medication. We defined diabetes as a single study visit with a fasting plasma glucose ≥7.0 mmol/L and/or non-fasting plasma glucose ≥11.1 mmol/L or receipt of diabetes medication. We computed descriptive statistics for hypertension/diabetes prevalence and treatment and used random intercept models adjusted for demographic and clinical characteristics to examine predictors of untreated hypertension and diabetes. From 2013 through 2023, among 3617 participants, 692 (19.1%) met our definition of hypertension, with a prevalence of 19.5% among PLWH and 17.3% among PLWoH; 276 (39.9%) received hypertension treatment. No significant difference in untreated hypertension was found comparing PLWH with PLWoH (adjusted risk ratio [aRR] 0.93, 95% confidence interval [CI]: 0.84–1.04). Among 3995 participants, 253 (6.3%) met our definition of diabetes, with a prevalence of 6.6% among PLWH and 4.7% among PLWoH; 51 (20.2%) received diabetes treatment. No significant difference in untreated diabetes was found comparing PLWH with PLWoH (aRR 1.01, 95% CI: 0.91–1.12). The high burden of untreated hypertension and diabetes among PLWH and PLWoH highlight the need for integrated non-communicable disease management within existing HIV services and strengthening of primary healthcare systems.

## Introduction

In African countries, non-communicable diseases (NCDs) are increasingly important contributors to morbidity and mortality [1], especially among people living with HIV (PLWH). The global scale-up of antiretroviral therapy (ART) has transformed HIV into a manageable lifelong condition, increasing time at risk for NCDs [2–4]. Living with HIV also independently contributes to NCD risk. For example, PLWH have approximately twice the risk of developing atherosclerotic cardiovascular disease compared with people living without HIV (PLWoH) [5], which may be related to HIV-specific mechanisms like immune activation and chronic inflammation [6]. ART may also contribute to NCD risk, for example, the global transition from efavirenz- to dolutegravir-containing ART regimens has been associated with excess weight gain, which may adversely impact blood pressure and lipids [7,8]. Furthermore, both increasing country-level HIV prevalence and ART use have been associated with higher prevalence of diabetes and obesity in African countries [9].

In 2019, the global prevalence of arterial hypertension was 32% and 34% among adult women and men, respectively. The global prevalence of diabetes was 9% and 10% among adult women and men, respectively [10,11]. Both hypertension and diabetes are leading contributors to global NCD morbidity and mortality. In 2017, high systolic blood pressure was associated with 10.4 million deaths and 218 million disability adjusted life years (DALYs) and high fasting plasma glucose was associated with 6.5 million deaths and 171 million DALYs [12]. Disability and mortality rates associated with hypertension and diabetes are among the highest in sub-Saharan African countries [13,14], emphasizing the need to improve diagnosis, treatment, and control in the region. In sub-Saharan Africa, of people with hypertension in 2019, an estimated 34% of men and 48% of women were diagnosed; of those diagnosed, 22% of men and 29% of women were treated; and of those on treatment, 9% of men and 13% of women had blood pressure controlled [10]. Furthermore, in the past 30 years, there has been little change in these outcomes in sub-Saharan African countries, whereas they have improved for most other countries [10]. The situation is similar for diabetes in sub-Saharan Africa, whereby the proportions of people diagnosed, treated, and with glycemic control are exceptionally low [15,16].

Effectively managing hypertension and diabetes among PLWH has especially high clinical and public health importance in sub-Saharan Africa where most PLWH reside [17]. Despite PLWH being frequently engaged in care with ART programs, several studies document major gaps in hypertension and diabetes diagnosis, treatment, and control [18–27], as also observed among general adult populations [10,15,16,28,29]. In sub-Saharan Africa, HIV care programs have historically been siloed, focusing exclusively on HIV testing, prevention, and treatment [30]. The United States President’s Emergency Plan for AIDS Relief (PEPFAR) supports HIV programming to millions of people globally and its strategic direction to end the HIV pandemic includes a focus on integrating HIV programming more efficiently into local health service delivery to provide person-centered care [31]. However, best practices for integrating NCD care with HIV testing, prevention, and treatment services have not yet been established and should be informed through an understanding of the local context [30]. We estimated treatment coverage for hypertension and diabetes among PLWH and PLWoH accessing services at PEPFAR-supported health facilities in Kenya, Nigeria, Tanzania, and Uganda and examined predictors of untreated hypertension and diabetes, including HIV status and other clinical and socio-demographic factors.

## Methods

### Study design and procedures

The prospective and ongoing African Cohort Study (AFRICOS) enrolls individuals aged ≥15 years who are receiving clinical care at 12 PEPFAR-supported facilities located at five programs in Kisumu West, Kenya; South Rift Valley, Kenya; Abuja & Lagos, Nigeria; Mbeya, Tanzania; and Kayunga, Uganda. Recruitment and enrollment began in January 2013 in Kayunga, Uganda; May 2013 in Abuja & Lagos, Nigeria; October 2013 in Kisumu West, Kenya; and November 2013 in South Rift Valley, Kenya and Mbeya, Tanzania. Recruitment/enrollment is ongoing across all program sites. The cohort primarily enrolls PLWH accessing treatment services and a smaller group of PLWoH accessing prevention and testing services.

At the enrollment visit and 6-monthly follow-up visits, participants undergo a physical exam, provide medical history (including all medications), and complete detailed demographic and behavioral questionnaires. At annual visits, phlebotomy for laboratory testing, including serum creatine and glucose, is conducted.

To ensure comparability of data across program sites, standard operating procedures for vital signs were followed, and laboratory measures were performed at laboratories that were accredited by the College of American Pathologists or had successfully completed external quality assurance. All data were recorded on paper case report forms and then entered into an electronic data capture system.

### Ethical considerations

The study was approved by institutional review boards of the Walter Reed Army Institute of Research, Makerere University School of Public Health, Kenya Medical Research Institute, TENWEK Hospital, Tanzania National Institute of Medical Research, Tanzania Ministry of Health, and Nigerian Ministry of Defence. All participants provided written informed consent prior to enrollment. Written informed consent was obtained from the parent/guardian as well as an informed assent for adolescents under 18 years of age. Emancipated or mature minors ages 15–17 years completed the standard informed consent.

### Definitions

Blood pressure was measured at every 6-monthly study visit. Repeat blood pressure measurements were taken for participants with elevated readings in Tanzania and Nigeria and for participants with either elevated or abnormally low readings in Uganda and Kenya. The availability of blood pressure cuff sizes varied by program site, with most program sites having access to standard cuffs for adults; however, larger blood pressure cuff sizes were available in Nigeria for participants who were obese or severely obese to ensure accuracy across body types. We defined hypertension as a systolic blood pressure (SBP) ≥140 mmHg at two consecutive study visits and/or a diastolic blood pressure (DBP) ≥90 mmHg at two consecutive study visits [32], or initiation of any blood pressure lowering medication. Plasma glucose was measured at annual study visits, with fasting status self-reported by the participant. We defined diabetes as a single study visit with a fasting plasma glucose 7.0 mmol/L (≥126 mg/dL) and/or non-fasting plasma glucose 11.1 mmol/L (≥200 mg/dL) [33], or receiving insulin or any oral or injectable diabetes medication. For both hypertension and diabetes, when a participant met these definitions, we considered them to have the condition for all subsequent study visits.

Our main outcomes were untreated hypertension and untreated diabetes among those meeting our definitions for hypertension or diabetes. Among individuals with hypertension and diabetes, we classified subsequent study visits as having received treatment or not, with hypertension treatment consisting of any blood pressure lowering medication (i.e., diuretics, beta-blockers, alpha blockers, alpha agonists, angiotensin receptor blockers [ARBs], angiotensin-converting enzyme [ACE] inhibitors, vasodilators) and diabetes treatment consisting of insulin or any diabetes medication (i.e., biguanides, sulfonylureas, GLP-1 receptor agonists, DPP-IV inhibitors, SGLT-2 inhibitors). In this definition, we only considered medications currently being taken by the participant at each visit and excluded medication that had been started and stopped between visits, to increase specificity to chronic hypertension/diabetes treatment. Among individuals receiving hypertension treatment, we compared regimens with current World Health Organization (WHO) guidelines that recommend 2 or more drugs from thiazide/thiazide-like diuretic, angiotensin-converting enzyme inhibitor or angiotensin II receptor blocker, dihydropyridine CCB [34].

At a study visit level, we defined blood pressure control as <130/80 mmHg [32] and defined glycemic control as fasting plasma glucose 80–130 mg/dL (4.4–7.2 mmol/L) or non-fasting plasma glucose <180 mg/dL (10.0 mmol/L) [35], both among individuals receiving treatment.

Our main predictor of interest was HIV status, which was defined at cohort enrollment and included people known to be living with HIV and PLWoH who consented to and underwent HIV testing and pre-/post-test counseling conducted at every six-monthly study visit. Other variables were selected based on their potential impact on receiving or accessing hypertension/diabetes treatment. Socio-demographic characteristics were based on self-reported responses to questionnaires: sex assigned at birth, age, program site (Kisumu West, Kenya; South Rift Valley, Kenya; Abuja & Lagos, Nigeria; Mbeya, Tanzania; Kayunga, Uganda), education level, employment status, quartiles of household income (income quartiles were first calculated separately for each country and then combined, i.e., the highest income quartile for Uganda was grouped with the highest income quartiles for Tanzania, Kenya and Nigeria, etc.), number of economic dependents (reported as the number of people the participant provides for economically), distance from the study facility, smoking status, alcohol use status, and recreational drug use. Clinical characteristics were body mass index (BMI) category (based on measured height and weight with <18.5 kg/m^2^ considered underweight and ≥25 kg/m^2^ considered overweight or obese), depression (Center for Epidemiological Studies Depression score ≥16) [36], food insecurity (self-report of not having enough food to eat over the past 12 months or receiving <3 meals/day) [37], and renal insufficiency (estimated glomerular filtration rate (eGFR) <60 mL/min/1.73m^2^ based on CKD-EPI 2021) [38]. Creatinine levels were assessed at six-monthly visits until 2020 and have since been assessed annually. Serum creatinine, age, and sex assigned at birth were used to calculate eGFR.

### Statistical analyses

For the hypertension analyses, we included all participants who had at least two study visits and for the diabetes analyses, we included all participants with at least one study visit with a plasma glucose measurement. We excluded study visits where female participants reported to be pregnant or had a positive pregnancy screening test to make our analyses more specific to essential hypertension and non-gestational diabetes. We described population characteristics overall and by hypertension/diabetes status. We described hypertension/diabetes medication use and quantified blood pressure and glycemic control by computing the frequency of treatment visits and the proportion of treatment visits with uncontrolled blood pressure and plasma glucose. We used random intercept log-Poisson models with robust standard errors to generate risk ratios and 95% confidence intervals (CIs) to examine associations between HIV status and other predictors with risk of untreated hypertension and diabetes, first running crude models and then multivariable models.

To address missingness introduced by differences in data collection procedures, i.e., some measures collected at each six-monthly visit while others collected annually, measures collected annually (plasma glucose; creatinine) were carried forward from the last annual visit where available. After this was done, any remaining missing data in regression models were handled using a complete case approach. An alpha level of 0.05 was used to determine statistical significance and analyses were done in SAS 9.4 (SAS Institute, Inc.; Cary, NC) and Stata version 18.0 (StataCorp, College Station, Texas).

## Results

From January 2013 through June 2023, 4114 individuals were enrolled in AFRICOS (S1 Fig). For the hypertension analyses, 3617 participants were included with a total of 16,579.7 person-years of follow-up and a median total duration of follow-up per participant of 6.2 (IQR 2.6–7.7) years. For the diabetes analyses, 3995 participants were included with a total of 18,086.1 person-years of follow-up and a median total duration of follow-up per participant of 5.6 (IQR 1.5–7.5) years. The only reason for excluding individual participants was not having ≥2 visits with systolic and/or diastolic blood pressure measurements (n = 497) and not having ≥1 visit with a plasma glucose measurement and/or fasting blood draw indicator (n = 119). In both hypertension and diabetes analyses, 487 visits among 343 female participants were excluded because of documented pregnancy. No participants were excluded entirely because of pregnancy. In both analyses, 84% of participants were PLWH.

### Hypertension treatment and control

**Fig 1:**
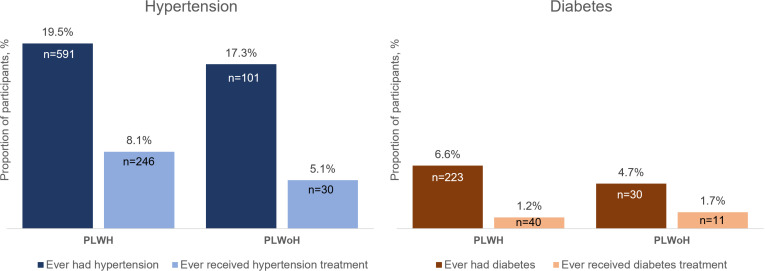
Hypertension and diabetes prevalence and treatment status among people living with and without HIV. PLWH, people living with HIV; PLWoH, people living without HIV. The bar graphs show the prevalence of hypertension and ever receipt of hypertension treatment (left) and prevalence of diabetes and ever receipt of diabetes treatment (right) per the definitions used during observation in the cohort and stratified by HIV status. The proportions are computed out of the total number of participants in the analytic populations for hypertension (PLWH n = 3,033; PLWoH n = 584) and diabetes (PLWH n = 3,362; PLWoH n = 633).

Of the 3617 participants included in the hypertension analyses, 692 (19.1%) met our definition of hypertension at any point during study follow-up, with 591 (19.5%) PLWH and 101 (17.3%) PLWoH (Table 1 and Fig 1). The overall crude incidence of hypertension was 36.2 per 1000 person-years (95% CI: 33.4–39.2) with 600 incident cases. Among PLWH, the incidence was 35.6 per 1000 person-years (95% CI: 32.7–38.9) with 505 incident cases and among PLWoH was 39.6 per 1000 person-years (95% CI: 32.4–48.5) with 95 incident cases (unadjusted hazard ratio for PLWH vs. PLWoH: 0.91; 95% CI 0.73–1.14; p = 0.417). Compared to those without hypertension, participants with hypertension were older (median age 47.6 vs. 40.1 years), more often at certain program sites (e.g., Mbeya, Tanzania and Abuja & Lagos, Nigeria), had a higher education level (e.g., 37.4% vs. 27.8% with a secondary education or higher), had a higher household income (e.g., 25.1% vs. 17.4% in highest income quartile), had more economic dependents (e.g., 35.3% vs. 30.5% with ≥6 economic dependents), resided farther from the study facility (e.g., 30.2% vs. 25.2% >20 km from facility), more often had food insecurity (34.0% vs. 24.0%), more often drank alcohol (18.2% vs. 12.2%), and were more often overweight or obese (51.2% vs. 29.6%) (Table 1).

**Table 1:** Characteristics of the analytic populations for hypertension and diabetes analyses.

	Hypertension			Diabetes		
	All (N = 3,617)	Hypertension (N = 692)	No hypertension (N = 2,925)	All (N = 3,995)	Diabetes (N = 253)	No diabetes (N = 3,742)
HIV status						
PLWH	3,033 (83.9%)	591 (85.4%)	2,442 (83.5%)	3,362 (84.2%)	223 (88.1%)	3,139 (83.9%)
PLWoH	584 (16.1%)	101 (14.6%)	483 (16.5%)	633 (15.8%)	30 (11.9%)	603 (16.1%)
Sex assigned at birth						
Male	1,527 (42.2%)	313 (45.2%)	1,214 (41.5%)	1,691 (42.3%)	126 (49.8%)	1,565 (41.8%)
Female	2,090 (57.8%)	379 (54.8%)	1,711 (58.5%)	2,304 (57.7%)	127 (50.2%)	2,177 (58.2%)
Age (years), median (IQR)^†^	41.8 (32.6–50.0)	47.6 (40.7–54.1)	40.1 (30.2–48.4)	41.1 (30.3–49.7)	47.0 (39.5–54.3)	40.5 (29.7–49.4)
Age (years)^†^						
<30	743 (20.5%)	28 (4.0%)	715 (24.4%)	969 (24.3%)	17 (6.7%)	952 (25.4%)
30–39	869 (24.0%)	134 (19.4%)	735 (25.1%)	918 (23.0%)	50 (19.8%)	868 (23.2%)
40–49	1,100 (30.4%)	248 (35.8%)	852 (29.1%)	1,132 (28.3%)	96 (37.9%)	1,036 (27.7%)
50+	904 (25.0%)	282 (40.8%)	622 (21.3%)	975 (24.4%)	90 (35.6%)	885 (23.7%)
Missing	1 (0.0%)	0 (0.0%)	1 (0.0%)	1 (0.0%)	0 (0.0%)	1 (0.0%)
Program site						
Kayunga, Uganda	623 (17.2%)	69 (10.0%)	554 (18.9%)	674 (16.9%)	28 (11.1%)	646 (17.3%)
South Rift Valley, Kenya	1,244 (34.4%)	263 (38.0%)	981 (33.5%)	1,370 (34.3%)	96 (37.9%)	1,274 (34.0%)
Kisumu West, Kenya	689 (19.0%)	84 (12.1%)	605 (20.7%)	727 (18.2%)	32 (12.6%)	695 (18.6%)
Mbeya, Tanzania	656 (18.1%)	160 (23.1%)	496 (17.0%)	737 (18.4%)	51 (20.2%)	686 (18.3%)
Abuja and Lagos Nigeria	405 (11.2%)	116 (16.8%)	289 (9.9%)	487 (12.2%)	46 (18.2%)	441 (11.8%)
Highest level of education^†^						
None or some primary	1,071 (29.6%)	195 (28.2%)	876 (29.9%)	1,149 (28.8%)	75 (29.6%)	1,074 (28.7%)
Primary or some secondary	1,459 (40.3%)	235 (34.0%)	1,224 (41.8%)	1,596 (39.9%)	91 (36.0%)	1,505 (40.2%)
Secondary and above	1,073 (29.7%)	259 (37.4%)	814 (27.8%)	1,198 (30.0%)	86 (34.0%)	1,112 (29.7%)
Missing	14 (0.4%)	3 (0.4%)	11 (0.4%)	52 (1.3%)	1 (0.4%)	51 (1.4%)
Currently employed^†^						
No	2,278 (63.0%)	410 (59.2%)	1,868 (63.9%)	2,544 (63.7%)	143 (56.5%)	2,401 (64.2%)
Yes	1,323 (36.6%)	279 (40.3%)	1,044 (35.7%)	1,398 (35.0%)	109 (43.1%)	1,289 (34.4%)
Missing	16 (0.4%)	3 (0.4%)	13 (0.4%)	53 (1.3%)	1 (0.4%)	52 (1.4%)
Quartiles of income by country^†^						
Lowest quartile	1,123 (31.0%)	192 (27.7%)	931 (31.8%)	1,262 (31.6%)	84 (33.2%)	1,178 (31.5%)
Second quartile	693 (19.2%)	119 (17.2%)	574 (19.6%)	762 (19.1%)	35 (13.8%)	727 (19.4%)
Third quartile	992 (27.4%)	202 (29.2%)	790 (27.0%)	1,079 (27.0%)	70 (27.7%)	1,009 (27.0%)
Highest quartile	684 (18.9%)	174 (25.1%)	510 (17.4%)	693 (17.3%)	60 (23.7%)	633 (16.9%)
Missing	125 (3.5%)	5 (0.7%)	120 (4.1%)	199 (5.0%)	4 (1.6%)	195 (5.2%)
Number of economic dependents^†^						
None	387 (10.7%)	23 (3.3%)	364 (12.4%)	502 (12.6%)	9 (3.6%)	493 (13.2%)
1 person	219 (6.1%)	33 (4.8%)	186 (6.4%)	253 (6.3%)	12 (4.7%)	241 (6.4%)
2–5 people	1,851 (51.2%)	387 (55.9%)	1,464 (50.1%)	2,029 (50.8%)	138 (54.5%)	1,891 (50.5%)
≥6 people	1,135 (31.4%)	244 (35.3%)	891 (30.5%)	1,149 (28.8%)	92 (36.4%)	1,057 (28.2%)
Missing	25 (0.7%)	5 (0.7%)	20 (0.7%)	62 (1.6%)	2 (0.8%)	60 (1.6%)
Distance from study facility^†^						
≤4 km	902 (24.9%)	151 (21.8%)	751 (25.7%)	1,022 (25.6%)	50 (19.8%)	972 (26.0%)
>4–10 km	887 (24.5%)	152 (22.0%)	735 (25.1%)	960 (24.0%)	65 (25.7%)	895 (23.9%)
>10–20 km	877 (24.2%)	179 (25.9%)	698 (23.9%)	958 (24.0%)	68 (26.9%)	890 (23.8%)
>20 km	947 (26.2%)	209 (30.2%)	738 (25.2%)	1,048 (26.2%)	70 (27.7%)	978 (26.1%)
Missing	4 (0.1%)	1 (0.1%)	3 (0.1%)	7 (0.2%)	0 (0.0%)	7 (0.2%)
Food insecure^†^						
No	2,669 (73.8%)	454 (65.6%)	2,215 (75.7%)	2,918 (73.0%)	164 (64.8%)	2,754 (73.6%)
Yes	936 (25.9%)	235 (34.0%)	701 (24.0%)	1,028 (25.7%)	88 (34.8%)	940 (25.1%)
Missing	12 (0.3%)	3 (0.4%)	9 (0.3%)	49 (1.2%)	1 (0.4%)	48 (1.3%)
Depression^†^						
Not depressed (score 0–15)	3,413 (94.4%)	641 (92.6%)	2,772 (94.8%)	3,669 (91.8%)	219 (86.6%)	3,450 (92.2%)
Depressed (score ≥16)	190 (5.3%)	48 (6.9%)	142 (4.9%)	273 (6.8%)	32 (12.6%)	241 (6.4%)
Missing	14 (0.4%)	3 (0.4%)	11 (0.4%)	53 (1.3%)	2 (0.8%)	51 (1.4%)
Smoker^†^						
No	3,534 (97.7%)	673 (97.3%)	2,861 (97.8%)	3,857 (96.5%)	243 (96.0%)	3,614 (96.6%)
Yes	68 (1.9%)	16 (2.3%)	52 (1.8%)	84 (2.1%)	8 (3.2%)	76 (2.0%)
Missing	15 (0.4%)	3 (0.4%)	12 (0.4%)	54 (1.4%)	2 (0.8%)	52 (1.4%)
Drinks alcohol^†^						
No	3,117 (86.2%)	563 (81.4%)	2,554 (87.3%)	3,386 (84.8%)	201 (79.4%)	3,185 (85.1%)
Yes	484 (13.4%)	126 (18.2%)	358 (12.2%)	555 (13.9%)	50 (19.8%)	505 (13.5%)
Missing	16 (0.4%)	3 (0.4%)	13 (0.4%)	54 (1.4%)	2 (0.8%)	52 (1.4%)
Uses recreational drugs^†^						
No	3,580 (99.0%)	684 (98.8%)	2,896 (99.0%)	3,907 (97.8%)	247 (97.6%)	3,660 (97.8%)
Yes	23 (0.6%)	5 (0.7%)	18 (0.6%)	36 (0.9%)	4 (1.6%)	32 (0.9%)
Missing	14 (0.4%)	3 (0.4%)	11 (0.4%)	52 (1.3%)	2 (0.8%)	50 (1.3%)
BMI, median (IQR)^†^	22.9 (20.2–26.5)	25.2 (22.2–29.1)	22.4 (20.0–25.8)	22.8 (20.1–26.3)	24.5 (21.4–29.1)	22.6 (20.1–26.1)
BMI category^†^						
Underweight	358 (9.9%)	35 (5.1%)	323 (11.0%)	390 (9.8%)	18 (7.1%)	372 (9.9%)
Normal	2,036 (56.3%)	302 (43.6%)	1,734 (59.3%)	2,275 (56.9%)	115 (45.5%)	2,160 (57.7%)
Overweight/obese	1,220 (33.7%)	354 (51.2%)	866 (29.6%)	1,288 (32.2%)	120 (47.4%)	1,168 (31.2%)
Missing	3 (0.1%)	1 (0.1%)	2 (0.1%)	42 (1.1%)	0 (0.0%)	42 (1.1%)
Renal insufficiency^†^						
No	3,310 (91.5%)	621 (89.7%)	2,689 (91.9%)	3,817 (95.5%)	238 (94.1%)	3,579 (95.6%)
Yes	113 (3.1%)	24 (3.5%)	89 (3.0%)	167 (4.2%)	15 (5.9%)	152 (4.1%)
Missing	194 (5.4%)	47 (6.8%)	147 (5.0%)	11 (0.3%)	0 (0.0%)	11 (0.3%)
Elevated blood pressure at single visit^†^						
No	NA	NA	NA	3,359 (84.1%)	172 (68.0%)	3,187 (85.2%)
Yes	NA	NA	NA	597 (14.9%)	81 (32.0%)	516 (13.8%)
Missing	NA	NA	NA	39 (1.0%)	0 (0.0%)	39 (1.0%)
Systolic blood pressure, median (IQR)^†^	118.0 (106.0–129.0)	143.0 (135.0–153.0)	112.0 (103.0–122.0)	117.0 (106.0–127.0)	121.0 (110.0–134.0)	116.0 (105.0–126.0)
Missing	0 (0.0%)	0 (0.0%)	0 (0.0%)	39 (1.0%)	0 (0.0%)	39 (1.0%)
Diastolic blood pressure, median (IQR)^†^	73.0 (66.0–82.0)	90.0 (84.0–97.0)	70.0 (64.0–78.0)	72.0 (65.0–80.0)	80.0 (70.0–87.0)	71.0 (65.0–80.0)
Missing	0 (0.0%)	0 (0.0%)	0 (0.0%)	39 (1.0%)	0 (0.0%)	39 (1.0%)
Diabetes						
No	3,436 (95.0%)	649 (93.8%)	2,787 (95.3%)	NA	NA	NA
Yes	181 (5.0%)	43 (6.2%)	138 (4.7%)	NA	NA	NA
Fasting plasma glucose (mg/dL)^†^^,^^*^	79.3 (71.9–88.3)	85.5 (77.8–95.5)	78.4 (70.3–86.5)	79.3 (72.1–89.5)	142.3 (129.7–172.8)	79.3 (71.0–86.5)
Non-fasting plasma glucose (mg/dL)^†^^,^^*^	79.3 (69.5–91.4)	88.3 (77.5–106.3)	77.5 (68.5–88.8)	79.1 (68.5–90.1)	316.2 (216.8–565.7)	77.5 (68.5–90.1)
Total time in cohort (years), median (IQR)	6.2 (2.6–7.7)	7.0 (5.5–8.1)	6.0 (2.0–7.5)	5.6 (1.5–7.5)	7.0 (5.2–8.2)	5.5 (1.2–7.5)

† Time-varying variable assessed when met hypertension/diabetes definition or most recent visit for those who never met definition of hypertension/diabetes.

* For those included in the hypertension table, n = 2,477 had fasting plasma glucose values, n = 749 had non-fasting plasma glucose values and n = 391 (10.8%) were missing whether plasma glucose was collected fasted or non-fasted.

IQR, interquartile range; PLWH, people living with HIV; PLWoH, people living without HIV.

Overall, 246 (8.1%) PLWH and 30 (5.1%) PLWoH received hypertension treatment (Fig 1). Of the 591 PLWH with hypertension, 345 (58.4%) did not receive treatment and of the 101 PLWoH with hypertension, 71 (70.3%) did not receive treatment. Of the 276 total participants receiving treatment, the most common hypertension regimen at the most recent study visit was a dihydropyridine calcium channel blocker (CCB) with a thiazide diuretic (52 [18.8%]) followed by a dihydropyridine CCB alone (46 [16.7%]; S1 Table). At their most recent study visit, 156 (56.5%) of those being treated for hypertension were receiving a WHO recommended combination therapy ranging from 51.0% (26 of 51) in Mbeya, Tanzania to 65.9% (29 of 44) in Abuja & Lagos, Nigeria. The 276 participants receiving hypertension treatment had a median of 4 (IQR 2–8) visits on hypertension treatment and a median of 3 (IQR 2–6) visits with uncontrolled blood pressure on treatment (Table 2). The median proportion of visits on treatment with uncontrolled blood pressure was 0.8 (IQR 0.4–1.0), which was similar among PLWH and PLWoH. The overall crude incidence of hypertension control among those on treatment was 202.6 per 1000 person-years (95% CI: 173.8–236.1; Table 3). Participants who achieved blood pressure control had a median of 1.0 (IQR 0.3–2.6) years from starting medication to control, with 1.0 (IQR 0.3–2.6) years for PLWH and 1.6 (IQR 0.2–2.1) years for PLWoH.

**Table 2:** Blood pressure and glycemic control at study visits among participants receiving treatment, overall and by HIV status.

	All participants	HIV status	
		PLWH	PLWoH
**Hypertension, n participants**	n = 276	n = 246	n = 30
Visits on treatment among those with hypertension, median (IQR)	4 (2–8)	4 (2–8)	3 (2–9)
Visits with uncontrolled blood pressure among those with hypertension on treatment, median (IQR)	3 (2–6)	3 (1–6)	3 (2–6)
Proportion of visits with uncontrolled blood pressure on hypertension treatment, median (IQR)	0.8 (0.4–1.0)	0.8 (0.4–1.0)	0.9 (0.5–1.0)
**Diabetes, n participants**	n = 51	n = 40	n = 11
Visits on treatment among those with diabetes, median (IQR)	2 (1–4)	2 (1–5.5)	2 (1–3)
Visits with uncontrolled plasma glucose among those with diabetes on treatment, median (IQR)	2 (1–4)	2 (1–5)	1 (1–2)
Proportion of visits with uncontrolled plasma glucose on diabetes treatment, median (IQR)	0.8 (0.3–1.0)	0.9 (0.2–1.0)	0.7 (0.3–1.0)

IQR, interquartile range; PLWH, people living with HIV; PLWoH, people living without HIV.

**Table 3:** Incidence of blood pressure and glycemic control among participants receiving treatment, overall and by HIV status.

	All participants	HIV status	
		PLWH	PLWoH
**Hypertension**			
Person-time (years)	809.6	735.8	73.8
Number of participants achieving blood pressure control	164	148	16
Incidence of control per 1000 person-years (95% CI)	202.6 (173.8–236.1)	201.2 (171.2–236.3)	216.9 (132.9–354.0)
**Diabetes**			
Person-time (years)	135.0	116.9	18.1
Number of participants achieving glycemic control	26	20	6
Incidence of control per 1000 person-years (95% CI)	192.6 (131.1–282.8)	171.1 (110.4–265.2)	330.9 (148.7–736.6)

Among 276 participants receiving hypertension treatment, 265 were included in the incidence of blood pressure control calculations (n = 6 excluded due to missing hypertension medication start date; n = 5 excluded where first time point controlled was the same date as hypertension medication was initiated). Among 51 participants receiving diabetes treatment, 48 were included in the incidence of glycemic control calculations (n = 2 excluded due to missing diabetes medication start date; n = 1 excluded where first time point controlled was the same date as diabetes medication was initiated).

CI, confidence interval; PLWH, people living with HIV; PLWoH, people living without HIV.

In the multivariable model, HIV status was not significantly associated with risk of untreated hypertension (PLWH vs. PLWoH: adjusted risk ratio [aRR] 0.93, 95% CI: 0.84–1.04; **Table 4**). Variables associated with higher risk of untreated hypertension were male sex (aRR 1.18, 95% CI: 1.08–1.28); all younger age groups compared with ≥50 years (e.g., 40–49 years: aRR 1.14, 95% CI: 1.06–1.24); and Kisumu West, Kenya (aRR 1.29, 95% CI: 1.12–1.48) compared with Kayunga, Uganda. Having a secondary or above education level vs. none/some primary school was associated with lower risk of untreated hypertension (aRR 0.89, 95% CI: 0.79–1.00).

**Table 4:** Regression models evaluating associations between HIV status and other predictors of untreated hypertension.

	Unadjusted models^*^		Adjusted model^*^^,^^†^	
	Unadjusted risk ratio	95% CI	Adjusted risk ratio	95% CI
**HIV status**				
PLWH	0.94	0.84–1.05	0.93	0.84–1.04
PLWoH	Ref		Ref	
**Sex assigned at birth**				
Female	Ref		Ref	
Male	**1.14**	**1.05–1.23**	**1.18**	**1.08–1.28**
**Age group**				
15–29 years	**1.26**	**1.12–1.43**	**1.35**	**1.14–1.60**
30–39 years	**1.17**	**1.07–1.29**	**1.19**	**1.09–1.31**
40–49 years	**1.10**	**1.02–1.19**	**1.14**	**1.06–1.24**
≥50 years	Ref		Ref	
**Program site**				
Kayunga, Uganda	Ref		Ref	
South Rift Valley Kenya	0.91	0.80–1.03	0.91	0.78–1.07
Kisumu West, Kenya	**1.29**	**1.15–1.45**	**1.29**	**1.12–1.48**
Mbeya, Tanzania	1.06	0.93–1.21	1.12	0.95–1.32
Abuja & Lagos, Nigeria	1.05	0.91–1.21	1.15	0.97–1.36
**Education level**				
None or some primary	Ref		Ref	
Primary and/or some secondary	0.97	0.90–1.06	0.95	0.87–1.03
Secondary and above	0.91	0.83–1.01	**0.89**	**0.79–1.00**
**Currently employed in the formal sector (yes vs. no)**	0.96	0.89–1.03	0.95	0.87–1.04
**Household income quartile**				
First (lowest)	Ref		Ref	
Second	1.05	0.99–1.12	1.05	0.99–1.11
Third	1.02	0.95–1.09	1.00	0.93–1.07
Fourth (highest)	0.97	0.89–1.07	0.97	0.89–1.06
**Number of economic dependents**				
None	Ref		Ref	
1 person	1.07	0.90–1.28	1.03	0.87–1.23
2–5 people	1.09	0.94–1.27	1.05	0.90–1.23
≥6 people	1.10	0.94–1.29	1.04	0.88–1.23
**Distance from study facility**				
≤4 km	Ref		Ref	
>4–10 km	1.02	0.92–1.14	0.99	0.89–1.09
>10–20 km	0.94	0.83–1.05	0.96	0.86–1.07
>20 km	0.96	0.86–1.07	1.00	0.90–1.11
**Food insecure (yes vs. no)**	1.03	0.97–1.09	0.96	0.91–1.01
**Depression (yes vs. no)**	0.99	0.92–1.07	0.98	0.90–1.06
**Current smoker (yes vs. no)**	1.08	0.87–1.34	1.10	0.89–1.36
**Drinks alcohol (yes vs. no)**	**1.11**	**1.03–1.21**	1.05	0.97–1.14
**Uses recreational drugs (yes vs. no)**	0.78	0.45–1.38	0.75	0.43–1.33
**BMI category, n (%)**				
Underweight	1.04	0.93–1.16	1.00	0.90–1.11
Normal	Ref		Ref	
Overweight or obese	0.95	0.89–1.02	1.01	0.94–1.08
**Renal insufficiency (yes vs. no)**	0.99	0.91–1.09	0.99	0.90–1.08
**Diabetes (yes vs. no)**	0.94	0.83–1.06	0.96	0.85–1.08
**Duration of follow-up in years**	**1.02**	**1.00–1.05**	**1.04**	**1.01–1.06**

* Random intercept models (log-Poisson with robust standard errors). Statistically significant results are bolded.

† Adjusted model includes all variables in the table, restricted to participants with complete case data (observations n = 5,103, groups n = 678). Among 5,362 treatment visits, there were 5,103 (95.2%) with complete case data. We tested for multicollinearity using the variable inflation factor and a cutoff value of 5; no variables were found to be collinear.

IQR, interquartile range; PLWH, people living with HIV; PLWoH, people living without HIV; BMI, body mass index; CI, confidence interval.

### Diabetes treatment and control

Of the 3995 participants included in the diabetes analyses, 253 (6.3%) met our definition of diabetes with 223 (6.6%) PLWH and 30 (4.7%) PLWoH (Table 2 and Fig 1). The crude incidence of diabetes was 10.2 per 1000 person-years (95% CI: 8.8–11.8) with 178 incident cases and was 10.7 per 1000 person-years (95% CI: 9.2–12.4) with 166 incident cases among PLWH and 6.4 per 1000 person-years (95% CI: 3.6–11.2) with 12 incident cases among PLWoH (unadjusted hazard ratio for PLWH vs. PLWoH: 1.60; 95% CI 0.89–2.87; p = 0.119). Compared with those who did not meet the diabetes definition, a greater proportion of participants with diabetes were male (41.8% vs. 49.8%), older (median age 47.0 vs. 40.5 years), more often at certain program sites (e.g., Abuja & Lagos, Nigeria), more often currently employed (43.1% vs. 34.4%), had a higher household income (e.g., 23.7% vs. 16.9% in highest income quartile), had more economic dependents (e.g., 36.4% vs. 28.2% with ≥6 economic dependents), more often had food insecurity (34.8% vs. 24.1%), more often had depression (12.6% vs. 6.4%), more often drank alcohol (19.8% vs. 13.5%), were more often overweight or obese (47.4% vs. 31.2%), and more often had an elevated blood pressure at a single visit (32.0% vs. 13.8%) (Table 2).

Overall, 40 (1.2%) PLWH and 11 (1.7%) PLWoH ever received diabetes treatment (Fig 1). Of the 223 PLWH with diabetes, 183 (82.1%) did not receive treatment and of the 30 PLWoH with diabetes, 19 (63.3%) did not receive treatment. Of the 51 participants receiving treatment, the most common diabetes regimen at the most recent study visit was metformin with a sulfonylurea (20 [39.2%] followed by metformin alone (16 [31.4%]; S2 Table). The 51 participants had a median of 2 (IQR 1–4) visits on diabetes treatment and a median of 2 (IQR 1–4) visits with uncontrolled plasma glucose on treatment (Table 2). The median proportion of visits on treatment with uncontrolled plasma glucose was 0.8 (IQR 0.3–1.0), which was similar among PLWH and PLWoH. The overall crude incidence of diabetes control among the 51 on treatment was 192.6 per 1000 person-years (95% CI: 131.1–282.8; Table 3). Participants who achieved glycemic control took a median of 1.0 (IQR 0.2–3.9) years from starting medication to control, with 1.0 (IQR 0.3–3.0) years for PLWH and 0.9 (IQR 0.1–3.9) years for PLWoH.

In the multivariable model, HIV status was not significantly associated with risk of untreated diabetes (PLWH vs. PLWoH: aRR 1.01, 95% CI: 0.91–1.12; Table 5). Variables associated with higher risk of untreated diabetes were all younger age groups compared with ≥50 years (e.g., 40–49 years: aRR 1.15, 95% CI: 1.07–1.25); and program site, with participants from all sites having higher risk compared with those from Kayunga, Uganda. Variables inversely associated with untreated diabetes were an education level of primary and/or some secondary vs. none/some primary school (aRR 0.92, 95% CI: 0.85–0.99); having concurrent hypertension (aRR 0.89, 95% CI 0.82–0.97); having 6 or more economic dependents as compared to none (aRR 0.92, 95% CI 0.84–1.00); and having a household income in the third or highest quartile as compared to the lowest income quartile.

**Table 5:** Regression models evaluating associations between HIV status and other predictors of untreated diabetes.

	Unadjusted models^*^		Adjusted model^*^^,^^†^	
	Unadjusted risk ratio	95% CI	Adjusted risk ratio	95% CI
**HIV status**				
PLWH	1.05	0.94–1.17	1.01	0.91–1.12
PLWoH	Ref		Ref	
**Sex assigned at birth**				
Female	Ref		Ref	
Male	1.01	0.93–1.09	1.06	0.98–1.15
**Age group**				
15–29 years	**1.13**	**1.01–1.26**	**1.13**	**1.01–1.27**
30–39 years	**1.20**	**1.11–1.29**	**1.17**	**1.09–1.26**
40–49 years	**1.12**	**1.03–1.22**	**1.15**	**1.07–1.25**
≥50 years	Ref		Ref	
**Program site**				
Kayunga, Uganda	Ref		Ref	
South Rift Valley Kenya	1.08	0.92–1.27	**1.18**	**1.01–1.36**
Kisumu West, Kenya	**1.23**	**1.07–1.42**	**1.31**	**1.13–1.53**
Mbeya, Tanzania	1.13	0.97–1.32	**1.26**	**1.08–1.48**
Abuja & Lagos, Nigeria	**1.23**	**1.06–1.41**	**1.35**	**1.15–1.60**
**Education level**				
None or some primary	Ref		Ref	
Primary and/or some secondary	0.94	0.88–1.01	**0.92**	**0.85–0.99**
Secondary and above	0.93	0.83–1.03	0.89	0.78–1.02
**Currently employed in the formal sector (yes vs. no)**	0.97	0.90–1.04	1.01	0.91–1.12
**Household income quartile**				
First (lowest)	Ref		Ref	
Second	0.99	0.94–1.05	0.97	0.92–1.03
Third	0.94	0.87–1.02	**0.92**	**0.86–1.00**
Fourth (highest)	**0.90**	**0.82–0.99**	**0.92**	**0.85–0.99**
**Number of economic dependents**				
None	Ref		Ref	
1 person	1.02	0.97–1.07	0.99	0.91–1.09
2–5 people	**0.94**	**0.89–1.00**	0.95	0.88–1.03
≥6 people	**0.89**	**0.81–0.97**	**0.92**	**0.84–1.00**
**Distance from study facility**				
≤4 km	Ref		Ref	
>4 to 10 km	1.03	0.93–1.14	1.06	0.97–1.17
>10 to 20 km	1.01	0.88–1.15	1.00	0.91–1.11
>20 km	1.00	0.88–1.13	1.00	0.90–1.12
**Food insecure (yes vs. no)**	1.04	0.99–1.10	0.99	0.93–1.04
**Depression (yes vs. no)**	1.06	0.99–1.12	1.02	0.95–1.09
**Current smoker (yes vs. no)**	**1.12**	**1.07–1.17**	1.08	0.97–1.21
**Drinks alcohol (yes vs. no)**	1.05	0.96–1.15	0.98	0.90–1.07
**Uses recreational drugs (yes vs. no)**	**1.12**	**1.07–1.16**	0.98	0.88–1.09
**BMI category, n (%)**				
Underweight	1.06	0.99–1.14	1.06	0.99–1.14
Normal	Ref		Ref	
Overweight or obese	0.98	0.91–1.05	1.05	0.97–1.12
**Renal insufficiency (yes vs. no)**	1.01	0.90–1.13	1.04	0.94–1.16
**Elevated blood pressure at a single visit (yes vs. no)**	**0.86**	**0.78–0.96**	**0.89**	**0.82–0.97**
**Duration of follow-up in years**	1.00	0.98–1.03	1.02	0.99–1.04

* Random intercept models (log-Poisson with robust standard errors). Statistically significant results are bolded.

† Adjusted model includes all variables in the table, restricted to participants with complete case data (observations n = 1,666, groups n = 250). Among 1,700 treatment visits, there were 1,666 (98.0%) with complete case data. We tested for multicollinearity using the variable inflation factor and a cutoff value of 5; no variables were found to be collinear.

IQR, interquartile range; PLWH, people living with HIV; PLWoH, people living without HIV; BMI, body mass index; CI, confidence interval.

## Discussion

We identified a high burden of hypertension and diabetes and low treatment coverage of both conditions in AFRICOS. Although, crude prevalence estimates for hypertension and diabetes among both PLWH and PLWoH were lower than Global Burden of Disease general population estimates [10,11], prevalence estimates among PLWH in AFRICOS were similar to those from systematic reviews and meta-analyses of studies from African countries [39,40]. Among both PLWH and PLWoH, most hypertension and diabetes went untreated. If treated, participants had a high proportion of study visits with uncontrolled blood pressure and/or plasma glucose. We did not find evidence to suggest that the gap in hypertension or diabetes treatment differed substantially by HIV status in this population. PLWH are generally more engaged in clinical care compared to their peers without HIV, as with universal ART they return to the clinic regularly for viral load testing and ART refills [41]. Due to greater contact with the healthcare system and prior research finding that accessing ART was positively associated with being treated for hypertension [42], we anticipated smaller treatment gaps in hypertension and diabetes treatment among PLWH compared with PLWoH. This lack of difference by HIV status might be related to PLWH engaging in care that is highly resourced and focused on HIV but may also reflect how the multifactorial barriers [43,44] to hypertension and diabetes care could be shared by both PLWH and PLWoH. The lack of apparent difference might also be related to participation in a cohort study where abnormal blood pressure and plasma glucose findings are routinely referred for clinical follow-up for all participants regardless of HIV status. Nevertheless, our findings highlight the importance of identifying, evaluating, and scaling up strategies that meet individuals’ diverse health needs, such as integrated NCD/HIV care for PLWH [45].

Our regression models helped identify characteristics of people who might be at higher risk of untreated hypertension and diabetes. Specifically, we found that program site, younger age, and lower education level were associated with higher risk for both untreated hypertension and diabetes, and males were at increased risk for untreated hypertension. Compared with the site in Kayunga, Uganda, risk of untreated hypertension was significantly higher in Kisumu West, Kenya and risk of untreated diabetes was significantly higher at all other sites. We would expect that the differences by site are highly susceptible to the local context of NCD care. Both Kayunga, Uganda and Kisumu West, Kenya are the only sites in AFRICOS where hypertension and diabetes care are now fully integrated into the ART clinic, which allows conditions to be managed simultaneously at a single clinic visit. However, even in settings where NCD care is integrated with HIV care, hypertension and diabetes outcomes may remain suboptimal [46]. Program site differences in hypertension and diabetes outcomes may also be related to factors beyond NCD/HIV care integration that could impact treatment access, such as unpredictable supply-chain issues leading to potentially frequent medication stock-outs [21].

Younger participants had significantly higher risk of untreated hypertension and diabetes compared with those aged ≥50 years, highlighting the importance of appropriately linking younger people to care and addressing their barriers to accessing and adhering to treatment, which may be different than people in older age groups. Higher education level was protective against untreated hypertension and diabetes; however, other socioeconomic variables like food insecurity were not significantly associated. These findings suggest underlying factors related to educational attainment like health literacy [47] could be influencing access to treatment, a potential target for interventions. Males had a higher risk of untreated hypertension compared with females, suggesting possible gender-specific barriers in accessing appropriate care as have been well documented within HIV care in African countries [48].

A strength of our study is that we used longitudinal data, which allowed us to examine receipt of treatment after abnormally high blood pressure and/or glucose. Furthermore, longitudinal data allowed us to define hypertension using multiple elevated blood pressure measurements, rather than a single elevated measurement. For a clinical diagnosis of hypertension, international guidelines recommend confirming an elevated blood pressure measurement at two to three clinic visits at one-to-four-week intervals after an initial elevated measurement and encourage out-of-clinic confirmation [32]. To adapt this definition to our cohort study, we defined hypertension as a persistently elevated systolic and/or diastolic blood pressure at two or more consecutive 6-monthly visits. However, measurement error may still be present, as lack of out-of-clinic blood pressure measurements precluded accounting for white coat hypertension and masked hypertension. In contrast, our definition of diabetes may be more susceptible to measurement error, which we defined based on a single elevated plasma glucose value and self-reported fasting/non-fasting status. Although international guidelines allow for diabetes to be diagnosed using a single blood sample, diagnosis requires two abnormal test results from the same sample [33], which was not done. Furthermore, we included people with a non-fasting plasma glucose ≥11.1 mmol/L (200 mg/dL) in our diabetes definition; however, international guidelines for diagnosis would require these participants to also have symptoms of hyperglycemia or exceed this cut-off at two hours after an oral glucose tolerance test. Future studies could address these limitations and more accurately assess diabetes status by measuring hemoglobin A1C, which provides a longer-term assessment of glycemia. For both hypertension and diabetes, prevalence estimates may be overestimated as medication use was included in their respective definitions, and some participants may have used blood pressure- or glucose-lowering medications for other indications (e.g., beta blockers for treatment of cardiac arrhythmias). Additional limitations include that self-reported measures, such as alcohol and drug use, may be influenced by social desirability bias. Lastly, AFRICOS participants comprise a population of highly virally suppressed, treatment experienced PLWH who are routinely engaged in care and may not reflect the broader population of PLWH in these settings. PLWoH in AFRICOS may also be different from the general population because they are engaged in a long-term cohort study. Considering these characteristics, the large treatment gaps observed in our analyses may be an underestimation of the true treatment gap in the general population in these and other African countries.

## Conclusions

We identified a high burden of untreated and uncontrolled hypertension and diabetes regardless of HIV status among participants in a long-term prospective cohort in four African countries. These findings highlight the importance of adapting lessons learned from the programmatic successes of HIV to hypertension and diabetes care that will ultimately strengthen primary healthcare systems. As with HIV, NCD care in resource-constrained settings needs public health approaches that allow widespread access to low-cost medications and engagement of civil society to bring political will to invest in care infrastructure [49]. Furthermore, this work emphasizes the need to identify and evaluate strategies to optimally address NCDs within existing HIV testing, prevention, and treatment services, such as through care integration.

## Supporting information

S1 Fig Inclusion and exclusion criteria for A) hypertension analyses analytic population and B) diabetes analyses analytic population. (DOCX)

S1 Table Hypertension medication classes at most recent study visit. (DOCX)

S2 Table Diabetes medications/classes at most recent study visit. (DOCX)

S1 Checklist PLOS human participants research checklist. .
(DOCX)
