# Supplementary material for: Gaps in hypertension and diabetes treatment among people living with and without HIV: Findings from a prospective cohort study in Kenya, Nigeria, Tanzania, and Uganda, 2013–2023
Source: PLOS Glob Public Health. 2025 Apr 29;5(4):e0004464. doi: 10.1371/journal.pgph.0004464 (PMC12040259; doi:10.1371/journal.pgph.0004464)
Supplement: S1 Fig — (DOCX) [file pgph.0004464.s001.docx]

**S1 Fig. Inclusion and exclusion for analytic populations for A) hypertension and B) diabetes analyses**

**
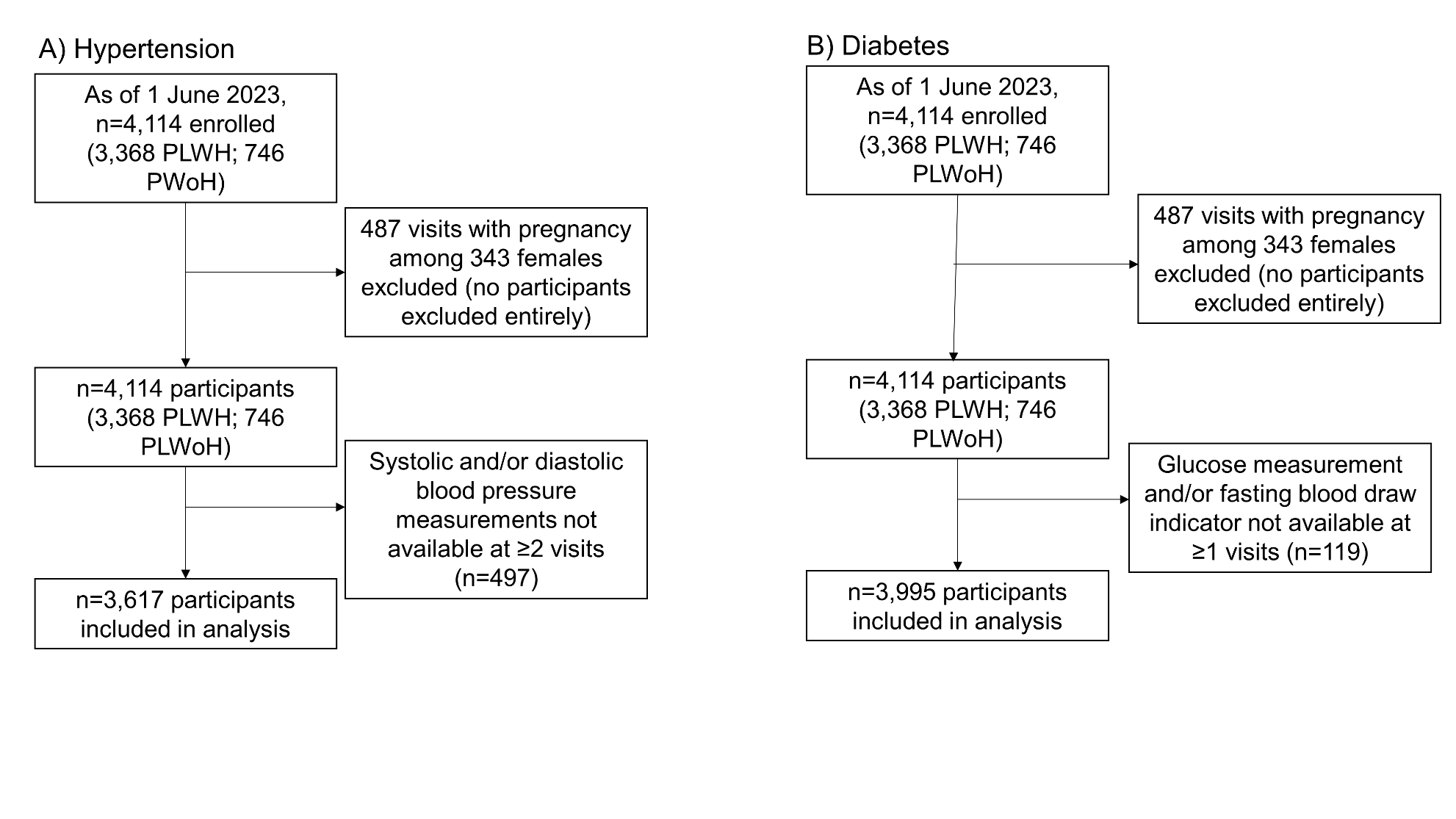
**

PLWH, people living with HIV; PLWoH, people living without HIV.
