# Supplementary material for: Gaps in hypertension and diabetes treatment among people living with and without HIV: Findings from a prospective cohort study in Kenya, Nigeria, Tanzania, and Uganda, 2013–2023
Source: PLOS Glob Public Health. 2025 Apr 29;5(4):e0004464. doi: 10.1371/journal.pgph.0004464 (PMC12040259; doi:10.1371/journal.pgph.0004464)
Supplement: S1 Table — (DOCX) [file pgph.0004464.s002.docx]

**S1 Table. Hypertension medication classes at most recent study visit**

| **Drug class** | **n** | **%** |
| --- | --- | --- |
| CCB + Thiazide | 52 | 19% |
| CCB | 46 | 17% |
| Thiazide | 24 | 9% |
| CCB + ACEi | 18 | 7% |
| Thiazide + ACEi | 15 | 5% |
| CCB + ARB | 14 | 5% |
| CCB + ACEi + Thiazide | 14 | 5% |
| CCB + ARB + Thiazide | 13 | 5% |
| Thiazide + ARB | 12 | 4% |
| Beta Blocker | 9 | 3% |
| Loop Diuretic | 8 | 3% |
| ARB | 8 | 3% |
| ACEi | 6 | 2% |
| CCB + Beta Blocker + Thiazide | 4 | 1% |
| CCB + Beta Blocker | 3 | 1% |
| Beta Blocker + Thiazide | 3 | 1% |
| ACEi + Loop Diuretic | 2 | 1% |
| CCB + ARB + Loop Diuretic | 2 | 1% |
| CCB + Thiazide + Potassium Sparing Diuretic | 2 | 1% |
| CCB + ACEi + Thiazide + Potassium Sparing Diuretic | 2 | 1% |
| Thiazide + Potassium Sparing Diuretic | 2 | 1% |
| Other | 17 | 6% |
| Total | 276 |  |

ACEi, angiotensin-converting enzyme inhibitor; ARB, angiotensin II receptor blocker; CCB, calcium channel blocker.
