# Supplementary material for: Gaps in hypertension and diabetes treatment among people living with and without HIV: Findings from a prospective cohort study in Kenya, Nigeria, Tanzania, and Uganda, 2013–2023
Source: PLOS Glob Public Health. 2025 Apr 29;5(4):e0004464. doi: 10.1371/journal.pgph.0004464 (PMC12040259; doi:10.1371/journal.pgph.0004464)
Supplement: S2 Table — (DOCX) [file pgph.0004464.s003.docx]

**S2 Table. Diabetes medications/classes at most recent study visit**

| **Drug class** | **n** | **%** |
| --- | --- | --- |
| Metformin + sulfonylurea | 20 | 39% |
| Metformin | 16 | 31% |
| Insulin | 9 | 18% |
| Other | 6 | 12% |
| Total | 51 |  |
