# Supplementary material for: Gaps in hypertension and diabetes treatment among people living with and without HIV: Findings from a prospective cohort study in Kenya, Nigeria, Tanzania, and Uganda, 2013–2023
Source: PLOS Glob Public Health. 2025 Apr 29;5(4):e0004464. doi: 10.1371/journal.pgph.0004464 (PMC12040259; doi:10.1371/journal.pgph.0004464)
Supplement: S1 Checklist — . (DOCX) [file pgph.0004464.s004.docx]

Inclusivity in global research

PLOS’ policy on inclusivity in global research aims to improve transparency in the reporting of research performed outside of researchers’ own country or community and ensures that PLOS publications reporting global research adhere to high standards for research ethics and authorship. Authors of relevant research articles may be asked to complete the questionnaire below, which outlines ethical, cultural, and scientific considerations specific to inclusivity in global research. This questionnaire may be requested when researchers have travelled to a different country to conduct research, if research uses samples collected in another country, research with Indigenous populations or their lands, or if research is on cultural artefacts. Researchers travelling to another country solely to use laboratory equipment will not normally be required to complete the questionnaire. However, the questionnaire can be requested at the journal’s discretion for any submission – if you have been requested to complete this questionnaire by the PLOS journal you submitted to, please do so.

Please complete the questionnaire below and include this as a Supporting Information file with your manuscript. Note that if your paper is accepted for publication, this checklist will be published with your article in the supporting information files. Please ensure that you reference the checklist in the main body of your manuscript. We suggest adding a subsection ‘Inclusivity in global research’ to your Methods section and adding the following sentence: “Additional information regarding the ethical, cultural, and scientific considerations specific to inclusivity in global research is included in the Supporting Information (SX Checklist)”

The questions have been designed to be applicable to a wide range of study types, and there are subsections for both human subjects research and non-human subjects research. If any of the questions are not relevant to your research please mark them as “N/A” as appropriate.

**Ethical considerations, permits and authorship**

*This section is applicable to all research types.*

Provide details as to who granted permissions and/or consent for the study to take place in the Methods section of your manuscript. This should include the names of **all** ethics boards, governmental organizations, community leaders or other bodies that provided approval for the study. If individuals provided approval refer to these people by their role or title but do not list their name(s).

Reported on page number: ***Ethical considerations are documented on page 6 of the manuscript.***

If there were any deviations from the study protocol after approval was obtained please provide details of these changes in the Methods section of your manuscript.

Reported on page number: ***Due to the extent of the AFRICOS study, including a duration of over 10 years and a large number of sites involved, we did not include a full list of deviations in the manuscript itself. If further requested by the journal, we are happy to provide a list of major and/or minor deviations. Please note that AFRICOS is an observational cohort study with minimal risk; procedures included in the protocol reflect standard of care in each setting.***

Did this study involve local collaborators that are residents of the country where the research was conducted or members of the community studied? If you do not have any authors from said communities, please provide an explanation for this below.

***Yes, this study involved local collaborators that are residents of the country where the research was conducted. At each program site, a*** ***local principal investigator oversees a team of trained clinical staff who conduct enrollment and follow-up study visits, administer questionnaires and conduct medical record abstraction. The local site principal investigators are included in the masthead authorship of this manuscript. Other local staff are recognized as members of the AFRICOS Study Group.***

Everyone listed as an author should meet PLOS’ criteria for authorship and all individuals who meet these criteria should be included in the author byline, rather than the acknowledgements. For further information please see the journal’s Authorship Policy.

**Human subjects research (e.g. health research, medical research, cross-cultural psychology)**

Did you obtain written informed consent from a representative of the local community or region before the research took place? How did you establish who speaks for the community? Details of written informed consent obtained from study participants should be reported separately in the Methods section of your manuscript.

***The local principal investigator(s) at each study site lead community engagement activities and communication with the local IRBs in each location*. In most countries, this includes a longstanding relationship with a community advisory board*. Relationships between the local AFRICOS site teams (located at: Kayunga Regional Referral Hospital, Kericho District Hospital, AC Litein Mission Hospital, Kapkatet District Hospital, Tenwek Mission Hospital, Kapsabet District Hospital, Nandi Hills District Hospital, Kisumu West District Hospital, Mbeya Zonal Referral Hospital, Mbeya Regional Referral Hospital, Defence Headquarters Medical Center, and the 68 Nigerian Army Reference Hospital) and the team at headquarters (Bethesda, MD) are longstanding and were established prior to initiation of the AFRICOS study. All participants provided written informed consent prior to any study procedures as described on page 6 of the manuscript.***

How did members of the local community provide input on the aims of the research investigation, its methodology, and its anticipated outcome(s)?

***AFRICOS is executed across multiple sites in four countries. All sites have community engagement staff that participate in study development and implementation. In Kenya, Uganda, and Tanzania, the sites have longstanding relationships with community advisory boards that provide input on the aims, methods, and outcomes of all studies conducted at those sites. The community advisory boards also facilitate dissemination of knowledge as research findings are generated.***

When engaging with the local community, how did you ensure that the informed consent documents and other materials could be understood by local stakeholders?

***Site principal investigators advised on the appropriate languages for use to reach target populations. Native speakers reviewed all informed consent documents and other participant-facing materials. All materials were reviewed and approved by local regulatory authorities, including translation and back-translation of any informed consent documents in languages other than English.***

Will the findings of the research be made available in an understandable format to stakeholders in the community where the study was conducted (e.g. via a presentation, summary report, copies of publications, etc.)? Please provide details of how this will be achieved.

***Yes, findings of this research and other work conducted under the AFRICOS protocol are distributed on a quarterly basis to stakeholders where the study is conducted, including to the site PIs and other clinical and research staff to whom the findings may be of interest or relevance. Findings are typically summarized in a powerpoint presentation for distribution and after peer review, copies of the full and final publication are distributed to all co-authors in pdf format, for further distribution as deemed appropriate. Community engagement officers and community advisory boards facilitate distribution to community members.***

**Non-human subjects research using specimens/ animals collected as part of the study, or those housed in archival collections. Examples include archaeology, paleontology, botany and zoology.**

Did the permission you obtained from a local authority to perform the study include an agreement on access to outputs and benefit sharing? This may include procedures to enable fair distribution of the benefits and resources arising from the research performed. Please include any details of Prior Informed Consent and Benefit Sharing Agreements obtained. These may be required by field-specific regulations, for example the Convention on Biological Diversity (CBD) and the associated Nagoya Protocol.

N/A

If the material used in your study was imported, please A) provide the year it was imported and B) indicate whether permits were obtained to import/export the materials used, C) provide details of any permits obtained. If this information is not available, please indicate this.

N/A

If you used archival specimens, please state how the material used in your study was acquired by the institute it is held in and provide details of any permits obtained for the original excavations/ sample collection. If this information is not available, please indicate this.

N/A

How was the potential cultural significance of the materials collected in your study to local communities considered in your research design? Were Indigenous peoples and/or local researchers and institutions involved with archaeological excavations / collection of specimens? If so, please provide a description of their involvement.

N/A

If your manuscript includes photographs of human remains please indicate whether authors obtained permission from descendants or affiliated cultural communities to do so.

N/A
